# Mining the Candidate Transcription Factors Modulating Tanshinones’ and Phenolic Acids’ Biosynthesis Under Low Nitrogen Stress in *Salvia miltiorrhiza*

**DOI:** 10.3390/ijms26041774

**Published:** 2025-02-19

**Authors:** Yating Cheng, Siqi Gui, Siyu Hao, Xiujuan Li, Chao Zhuang, Yifei Shi, Wei Zhou, Guoyin Kai

**Affiliations:** Laboratory for Core Technology of TCM Quality Improvement and Transformation, School of Pharmaceutical Sciences, Academy of Chinese Medical Science, Zhejiang Chinese Medical University, Hangzhou 310053, China

**Keywords:** *Salvia miltiorrhiza*, transcriptome sequencing, tanshinones, phenolic acids, low nitrogen stress, transcription factors

## Abstract

Mining valuable genes is helpful to breed high-quality *Salvia miltiorrhiza* exhibiting efficient nitrogen fertilizer utilization efficiency. In the present study, transcriptome sequencing was introduced to select the candidate transcription factors (TFs) involved in tanshinones’ (TAs) and phenolic acids’ (PHAs) biosynthesis as well as low nitrogen (LN) stress. In totally, 97.71 Gb clean data was obtained from fifteen sequencing samples and 30,975 unigenes were assembled. Among of them, 27,843 unigenes were successfully annotated. Overall, 8663 differential expression genes (DEGs) were identified, among of which 5034 unigenes were up-regulated, and 3629 unigenes were down-regulated. By enrichment of DEGs together with gene co-expression network construction, 10 candidate TFs including *HSFB2b*, *LBD12*, *ERF1A*, *ERF98*, *LBD25*, *HSF24*, *RAM1*, *HSFA4B*, *TCP8*, and *WRKY24* were finally retrieved, which are predicted to participate in modulating TA and PHA biosynthesis under LN stress. Quantitative real-time polymerase chain reaction (qRT-PCR) detection was introduced to further detect the expression profile of candidate TFs under LN stress. These findings offer a valuable resource for in-depth study of TAs ‘and PHAs’ biosynthesis under LN stress in *S. miltiorrhiza*.

## 1. Introduction

*Salvia miltiorrhiza*, also known as red sage or Danshen, is one of the most commonly used herbs in China [1,2]. Tanshinones (TAs) and phenolic acids (PHAs) are the major medicinal metabolites in *S. miltiorrhiza*, and they are validated to have numerous therapeutic effects such as anti-atherosclerotic, anti-hypertension, anti-osteoporosis and anti-tumor activities [1,2,3,4]. A high-quality germplasm resource of *S. miltiorrhiza* is scarce due to its overexploitation. Therefore, it is imperative to cultivate premium varieties of *S. miltiorrhiza* based on modern biotechnology.

The biosynthetic pathways of TAs and PHAs have been basically clarified. TAs are fat-soluble diterpenoids that are produced by two distinct biosynthetic pathways: the mevalonate (MVA) pathway and the 2-C-methyl-D-erythritol-4-phosphate (MEP) pathway [5,6,7,8,9]. In the MVA pathway, terpene is biosynthesized from the primary precursor acetyl-CoA to form the intermediate precursor isopentenyl diphosphate (IPP). In the MEP pathway, dimethylallyl diphosphate (DMAPP) is synthesized from glycolysis products glyceraldehydyde-3-phosphate and pyruvate through seven catalytic reactions. IPP and DMAPP are catalyzed by geranylgeranyl pyrophosphate synthase (GGPPS) to form geranylgeranyl pyrophosphate (GGPP). GGPP is subjected to a series of enzymatic reactions, catalyzed by either diterpenoid synthase or cyclase and multiple CYP450s, which ultimately results in the synthesis of the final product of TAs. However, the downstream conversion reaction from ferruginol to tanshinone is still not clear. PHAs are synthesized from phenylpropane and tyrosine pathways [10,11]. In the tyrosine biosynthetic pathway, L-tyrosine is catalyzed to form 3,4-dihydroxyphenolactic acid by two catalyzing enzymes. In the phenylpropane pathway, L-phenylalanine generates 4-coumaroyl-coenzyme A through three catalytic reaction steps. The above two intermediates, 3,4-dihydroxyphenyllactic acid and 4-coumaroyl-coenzyme A, undergo further modification by rosmarinic acid synthetase (RAS) and the P450 enzyme CYP98A14 to produce rosmarinic acid (RA), which is then conversed by several unknown enzymatic reactions to form phenolic acid A and B. Discovering and identifying the candidate transcription factors (TFs) targeting the above rate-limiting enzyme genes in the biosynthetic pathway of TAs and PHAs is beneficial to genetic improvement of *S. miltiorrhiza* varieties through genetic engineering technology.

Nitrogen nutrition plays a vital role in plant growth, development, yield, and primary and secondary metabolites’ biosynthesis [12,13,14]. The high utilization efficiency of nitrogen fertilizer is beneficial for reducing costs and relieving environmental stress in agricultural production. It has demonstrated that TFs play an irreplaceable role in regulating plant nitrogen uptake, growth, development, yield, etc. Overexpressing *OsNRT1.1A* can elevate nitrogen utilization efficiency and early flowering [13]. In rice (*Oryza sativa* L.), *OsSNAC1* was identified as an upstream regulator of the nitrate transporter gene *OsNRT2.1*. which can be up-regulated by *OsSNAC1*, thereby enhancing nitrogen utilization efficiency, growth and yield [15]. In *Atropa belladonna*, a bHLH TF was identified to positively regulate the tropane alkaloids’ biosynthesis in response to LN stress [16]. LN stress can also promote sterol and withaferin A biosynthesis in *Withania somnifera* (L.), of which it is validated to be mediated by a jasmonate-responsive WRKY transcription factor [17]. Therefore, elevating the utilization efficiency of nitrogen is crucial to increase plant yield and promote metabolites’ biosynthesis in production. But this research item is relatively poor in *S. miltiorrhiza*.

Many TFs not only improve nitrogen uptake but also enhance the biosynthesis of secondary metabolites in many plant species. It is verified that under low nitrogen (LN) stress, the BR-responsive transcription factor BZR1 promotes anthocyanin biosynthesis by interacting with PAP1/2 in *Arabidopsis thaliana* [18]. *bHLH130* not only regulates flavonoid and lignin biosynthesis derived from two parallel metabolic pathways but also improves the nitrogen uptake efficiency in apples [19]. However, little studies on how candidate TFs regulate TAs’ and PHAs’ biosynthesis in response to LN stress have been reported in *S. miltiorrhiza* till now.

Transcriptome sequencing has provided a valid research strategy for efficiently mining candidate genes, and it was also widely used to investigate the expression profiles of a large number of genes and identify potential functional genes in the whole genome of plants [20,21]. Based on the transcriptome database of yam tubers from four developmental stages, the candidate genes involved in post-harvest hardening (PHH) have been mined, of which it offers a valuable research basis for breeding new resistant varieties against PHH [22]. Combining the transcriptomics and metabolomics database, the candidate TFs related to nitrogen uptake have been systematically mined in *Tinospora sagittata*. This provides many important regulatory genes for breeding new varieties to elevate nitrogen fertilizer use efficiency and improve the medicinal substances’ biosynthesis in *T. sagittata* [23].

In *S. miltiorrhiza*, many studies have reported that TAs’ and PHAs’ biosynthesis can be regulated by diverse TFs or by plant growth regulators such as abscisic acid (ABA), methyljasmonic acid (MeJA), and salicylic acid (SA) mediated by certain TFs [24,25,26]. Certain miRNAs, DNA methylation modification, and protein phosphorylation have also been validated to effectively regulate TAs’ biosynthesis [27,28]. Mining valid genes to breed high-quality *S. miltiorrhiza* exhibiting efficient nitrogen fertilizer utilization efficiency is a valuable research item in production, but this work is poor. In the present study, high throughput sequencing technology was employed to examine differential expression TFs in *S. miltiorrhiza* hairy roots in response to LN stress. Gene co-expression analysis and qRT-PCR detection were independently introduced to mine the candidate TFs related to TAs’ and PHAs’ biosynthesis under LN stress in *S. miltiorrhiza*. It lays the research foundation to dissect the molecular regulatory network of candidate TFs to improve nitrogen uptake and medicinal substances’ biosynthesis, of which it is also beneficial to cultivate new germplasm resources of *S. miltiorrhiza* through genetic engineering manipulation.

## 2. Results

### 2.1. LN Stress Decreases TAs and PHAs Accumulation

**Figure 1:**
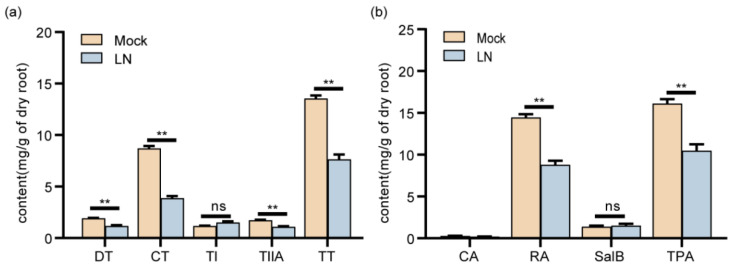
Content of TAs (**a**) and PHAs (**b**) in *S. miltiorrhiza* hairy roots under LN stress. DT, Dihydrotanshinone; CT, cryptotanshinone; TI, tanshinone I; TIIA, tanshinone IIA; TT, total of TAs. CA, caffeic acid; RA, rosmarinic acid; SalB, salvianolic acid B; TPA, total of PHAs. Error bars represent SD (n = 3), ** *p* < 0.01.

To investigate whether nitrogen affects the accumulation of TAs and PHAs, *S. miltiorrhiza* hairy roots were induced with LN stress. The results demonstrated that compared with the Mock, tanshinones (DT, CT, and TIIA) and total of tanshinones (TT) decreased after exposure to 7d of LN stress, while RA and total of phenolic acids (TPA) also exhibited a decreasing trend (Figure 1). In conclusion, LN stress inhibits the accumulation of TAs and PHAs in *S. miltiorrhiza* hairy roots.

### 2.2. Transcriptome Sequencing and De Novo Assembly

To further mine the candidate transcription factors (TFs) involved in TAs’ and PHAs’ biosynthesis under LN stress, transcriptome sequencing was employed to select DEGs in the hairy roots of *S. miltiorrhiza* after the induction of LN stress. A total of 97.71 Gb of clean data was obtained. Among this, the amount of clean data of each sample was more than 6.03 Gb and the average GC content was 48.72% (Table 1). After filtering adapter sequences, low quality, and short reads, an average of 6.51 Gb of clean reads was obtained and assembled using the Trinity software (version 2.2.3) to generate 30,975 unigenes (Table S1). The clean reads of each sample were all aligned with the reference genome of *S. miltiorrhiza*, with the alignment rates ranging from 91.6% to 93.13% (Table S2). The coverage of sequencing and length distribution of transcripts indicate that the quality of transcriptome sequencing is fulfilled with data mining (Figure S1a,b). Principal component analysis (PCA) was employed for further evaluating data stability, by which it exhibited stable reproducibility for the biological replicates (Figure S1c).

### 2.3. Functional Annotation and Classification of Unigenes

**Figure 2:**
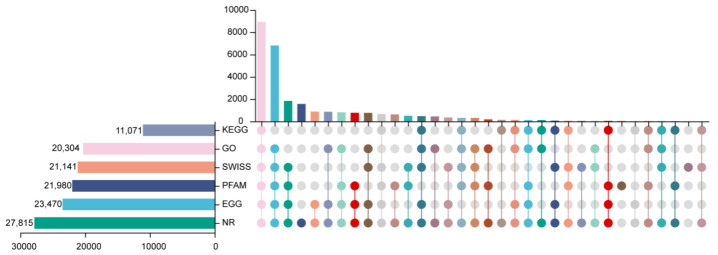
Annotation of unigenes. The bar chart on the left denotes the total number of unigenes; the connecting line represents annotated unigenes; and the number above indicates the number of unigenes annotated in the databases.

To predict the underlying function of all unigenes, 27,843 out of 30,975 unigenes (89.89%) were functionally annotated through blasting the six public protein databases. Of these, 27,815 (89.8%) unigenes in NR, 20,304 (65.55%) unigenes in GO, 23,470 (75.77%) unigenes in EggNOG, 21,980 (70.96%) unigenes in pfam, 21,141 (68.25%) unigenes in Swiss-Prot, and 11,071 (35.74%) unigenes in KEGG databases were annotated, respectively (Figure 2). GO enrichment comprises three categories: biological process (BP), cellular component (CC), and molecular function (MF), which were further subgrouped into 40 subcategories in level 2 GO term annotation. In the BP GO category, cellular processes accounted for the largest proportion, followed by metabolic processes, biological regulation, and response to stimulus. In the CC GO category, cellular anatomical entities followed by protein-containing complexes were the most enriched subcategories. In the MF GO category, binding was the highest represented category followed by catalytic activity and transporter activity (Figure S2a). To further explore the potential function of unigenes in *S. miltiorrhiza*, all the assembled unigenes were mapped against the KEGG database, which resulted in the identification of 20 main metabolic pathways (Figure S2b). Among them, carbohydrate metabolism, amino acid metabolism, lipid metabolism, and metabolism of terpenoids and polyketides were the four most represented pathways.

### 2.4. Differential Expression Gene and Enrichment Analysis

**Figure 3:**
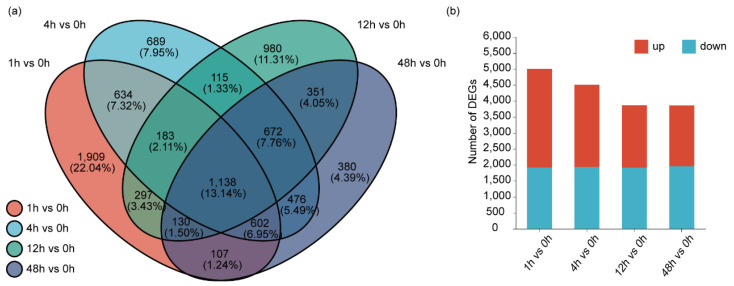
DEGs in response to LN stress. (**a**) Venn diagram for DEGs compared with each of the LN-induced time points (1, 4, 12 and 48 h) and the Mock (0 h), respectively. (**b**) The gene expression level under LN treatment at 1, 4, 12 and 48 h compared with the Mock (0 h), respectively.

**Figure 4:**
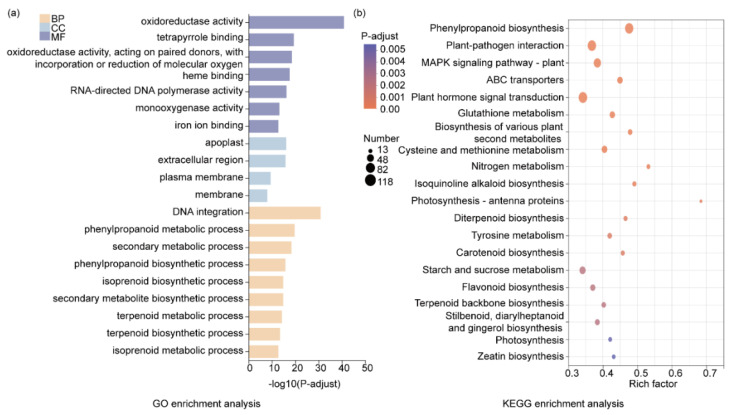
Functional enrichment analysis of DEGs. (**a**) GO enrichment analysis of DEG. The y-axis represents the GO terms, and the x-axis represents the level of significance of enrichment. The larger the log_10_ (*p*-adjust) value, the more significantly enriched the GO term is. (**b**) KEGG enrichment analysis of DEGs. The y-axis represents the KEGG pathway and the x-axis represents rich factor values; the size of the circles denotes the gene number. DEGs were mined using DESeq2 (version 1.24.0), with |log_2_FC| ≥ 1 and *p*-adjust < 0.05 (DESeq2) as the cutoff.

The normalized expression values of Transcripts Per Million mapped reads (TPM) of each unigene were calculated, and the fold change between the Mock (0 h) and induction groups (induced time points at 1, 4, 12, and 48 h, respectively) > 2-fold and *p*-adjust < 0.05 was set as the cutoff to choose the DEGs. Overall, 8663 DEGs were identified, among which 5034 unigenes were up-regulated, and 3629 unigenes were down-regulated (Table S3). In total, 1138 unigenes were labeled as DEGs at each induced time point vs. the Mock (Figure 3a). It is noteworthy that the largest number of DEGs was observed under the treatment of LN stress for 1 h vs. 0 h, with a total of 5000 genes identified as DEGs (Figure 3b). GO enrichment analysis was performed to annotate the DEGs. In the cellular component (CC) category, the extracellular region and apoplast were the most enriched subcategories. In the molecular function (MF) category, the enrichment of oxidoreductase activity was the highest, followed by tetrapyrrole binding, heme binding, and oxidoreductase activity, acting on paired donors, with incorporation of or reduction in molecular oxygen. In the biological process (BP) category, DNA integration, phenylpropanoid metabolic process, secondary metabolic process, phenylpropanoid biosynthetic process, isoprenoid biosynthetic process, and secondary metabolite biosynthetic process were the abundant enrichments (Figure 4a, Table S4). KEGG enrichment analysis revealed that nitrogen metabolism and phenylpropanoid biosynthesis were the most significantly enriched pathways, followed by biosynthesis of various plant secondary metabolites and terpenoid backbone biosynthesis (Figure 4b, Table S5). As two pathways of terpenoid backbone biosynthesis and phenylpropanoid biosynthesis are related to TAs’ and PHAs’ biosynthesis, this indicates that LN might regulate TAs’ and PHAs’ biosynthesis.

### 2.5. Differential Expression Genes Involved in TAs’ and PHAs’ Biosynthetic Pathway

**Figure 5:**
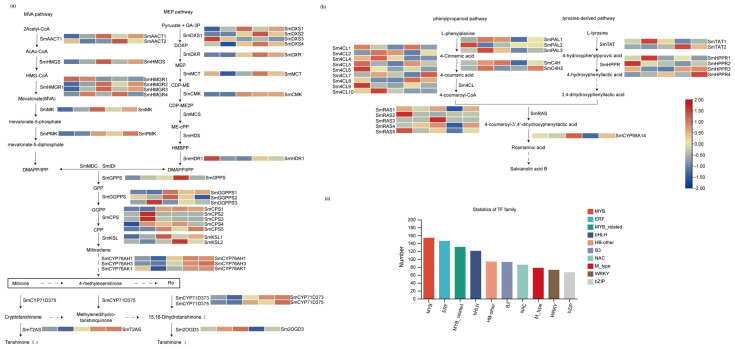
Gene expression and classification of transcription factors. (**a**) Expression profiles of TAs’ biosynthetic genes. (**b**) Expression profiles of PHAs’ biosynthetic genes. (**c**) The top 10 TF families with more than 65 members are listed. Solid arrows represent the confirmed synthetic pathways, while dashed arrows indicate intermediate processes that are uncertain and have not yet been resolved in the synthesis pathway.

Based on the transcriptome dataset, 38 unigenes encoding 24 synthetase genes involved in TAs’ biosynthetic pathway were found to show significant differential expression between the LN group and the Mock (0 h). Among them, 12 unigenes encoding SmHMGS, SmHMGR2, SmDXS1, SmDXR, SmGGPPS1, SmCPS1, SmKSL1, SmCYP76AH1, SmCYP76AH3, SmCYP71D373, and SmCYP71D375 were significantly up-regulated, whereas only 2 unigenes encoding SmCYP76AK1 and Sm2OGD3 exhibited a down-regulated expression pattern (Figure 5a, Table S6). In PHAs’ biosynthetic pathway, a total of 26 unigenes encoding 7 synthetase genes displayed differential expression, among which Sm4CL2, SmRAS1, SmHPPR1, and SmTAT1 were all up-regulated, while SmTAT1 had the highest induced expression level under the LN treatment and peaked at 1 h induction with a 2.57-fold increase compared to the Mock (Figure 5b, Table S7).

### 2.6. Differential Expression Profile of TF Family

To explore candidate TFs that regulate TAs’ and PHAs’ biosynthesis under the induction of LN, a total of 1551 unigenes encoding putative TFs in the *S. miltiorrhiza* transcriptome were annotated and further divided into 48 TF families, among which MYB families had the largest number, followed by ERF, MYB-related, bHLH, and HB TF families (Figure 5c). By DEG analysis, a total of 571 of 1551 TF unigenes were found to be differentially expressed between LN-induced groups (1, 4, 12, and 48 h) and the Mock (0 h). Among them, 325 TF unigenes were significantly up-regulated (Table S8), of which the ERF family represents the largest group, followed by WRKY, bHLH, MYB, and NAC families (Figure S3a). Among the down-regulated expressed TFs, the MYB family members achieved the largest number, followed by the MYB-related and ERF families (Figure S3b).

### 2.7. Transcription Factors Involved in LN Stress

**Figure 6:**
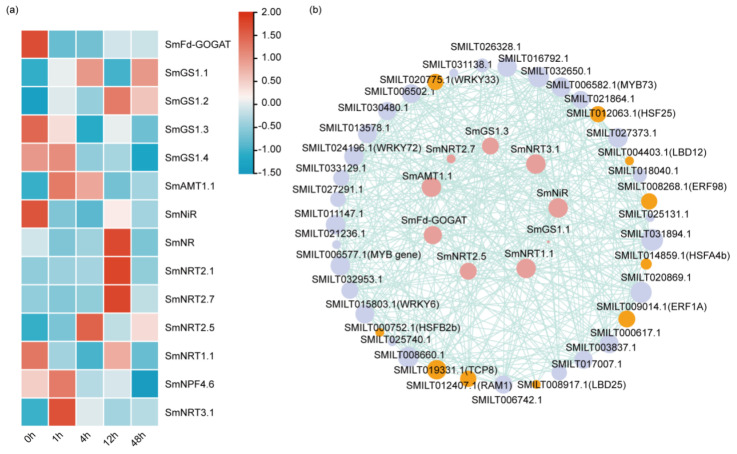
DEGs involved in LN stress. (**a**) Expression profiles of marker genes related to nitrogen uptake. (**b**) Co-expression network between differentially expressed marker genes involved in nitrogen uptake and candidate TFs. The light purple circles represent transcription factors with a co-expression coefficient greater than 0.9; the orange ones are the final ten selected transcription factors; and the rose-pink circles represent nitrogen-related genes.

By DEG analysis, 14 genes including *Fd-GOGAT* (ferredoxin-dependent glutamate synthase, *Fd-GOGAT*) [29], *GS1.1* (glutamine synthetase 1.1, *GS1.1*), *GS1.2* (glutamine synthetase 1.2, *GS1.2*), *GS1.3* (glutamine synthetase 1.3, *GS1.3*), *GS1.4* (glutamine synthetase 1.4, *GS1.4*), *AMT1.1* (ammonium transporter 1.1, *AMT1.1*), *NiR* (nitrite reductase, *NiR*), *NR* (nitrate reductase, *NR*), *NRT2.1* (nitrate transporter 2.1, *NRT2.1*), *NRT2.7* (nitrate transporter 2.7, *NRT2.7*), *NRT2.5* (nitrate transporter 2.5, *NRT2.5*), *NRT1.1* (peptide transporter family/nitrate transporter 1.1, *NRT1.1*), *NPF4.6* (peptide transporter family 4.6, as known as *NRT1.2*), and *NRT3.1* (nitrate transporter 3.1, *NRT3.1*, as known as *NAR*) related to nitrogen uptake were mined. Among them, seven of fourteen were up-regulated, while four members were drastically down-regulated (Figure 6a, Table S9). To explore the candidate TFs involved in LN stress, a gene co-expression network was constructed using 325 up-regulated DEGs encoding putative TFs and the above 11 genes associated with nitrogen uptake. The nodes of the network were gene combinations with Pearson correlation coefficients > 0.90. The co-expression network contained 38 nodes and 49 links. All pairs of TFs were positively correlated with nine of the fourteen genes involved in nitrogen uptake (Figure 6b, Table S10). Among the 38 positively correlated TFs, *WRKY33* (SMILT020775.1) had the highest relatedness, with the correlation coefficient reaching 0.98, followed by *MYB73* (SMILT006582.1), *WRKY6* (SMILT015803.1), *MYB gene* (SMILT006577.1), and *WRKY72* (SMILT024196.1), with *A. thaliana* or other medicinal plants named as the reference [30,31,32].

### 2.8. Transcription Factors Involved in TAs’ and PHAs’ Biosynthesis

**Figure 7:**
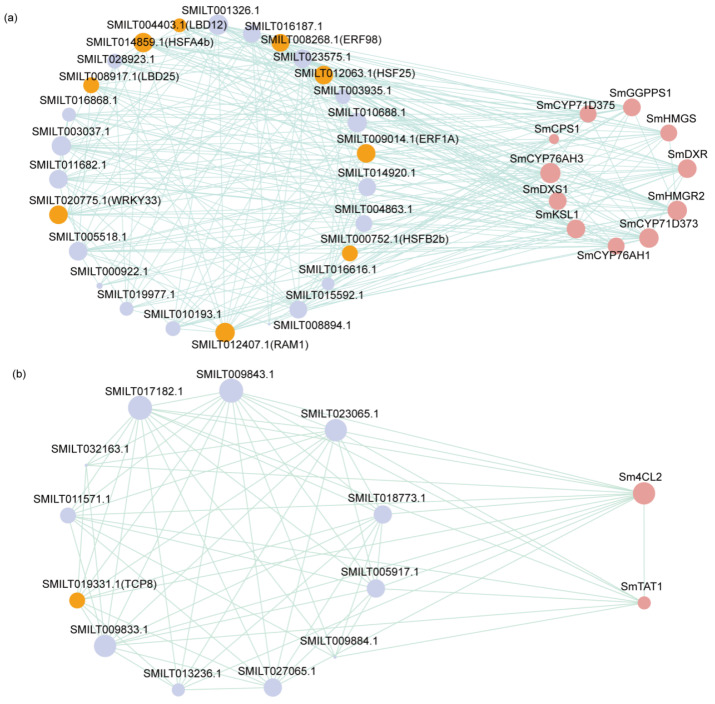
Co-expression relationship of candidate TFs and synthetase genes involved in TAs’ and PHAs’ biosynthesis. (**a**) Co-expression relationship of candidate TFs with synthetase genes related to TAs’ biosynthesis. (**b**) Co-expression relationship of candidate TFs with synthetase genes related to PHAs’ biosynthesis.The light purple circles represent transcription factors with a co-expression coefficient greater than 0.9; the orange ones are the final ten selected transcription factors; and the rose-pink circles represent differentially expressed synthases.

To select the candidate TFs participating in TAs’ and PHAs’ biosynthesis, 325 up-regulated DEGs encoding putative TFs and the biosynthetic genes of TAs and PHAs were employed to construct the gene co-expression network. The Pearson correlation coefficient was set > 0.9 and *p*-adjust < 0.05 as the cutoff to choose the candidate gene combinations in the nodes of each network. The network of TFs’ and TAs’ biosynthetic genes contained 27 nodes and 37 connections, with SMILT009014.1 (*ERF1A*) achieving the highest correlation coefficient of 0.975 with TAs’ biosynthetic genes, followed by SMILT012407.1 (*RAM1*), SMILT008268.1 (*ERF98*), and SMILT014859.1 (*HSFA4b*). Of these, their correlation coefficients were all more than 0.95 (Figure 7a, Table S11). Nevertheless, 12 nodes and 12 connections were found in the gene co-expression network of TAs’ and PHAs’ biosynthetic genes (Figure 7b, Table S12). The above result implies that the selected 39 TFs may play an important role in modulating TAs’ and PHAs’ biosynthesis in *S. miltiorrhiza*.

### 2.9. Mining the Candidate TFs Modulating TAs’ and PHAs’ Biosynthesis Under LN Stress

To screen candidate TFs that can simultaneously regulate medicinal substances’ biosynthesis and intensively correspond to LN induction in *S. miltiorrhiza* hairy roots, 39 candidate TFs that regulate TAs’ or PHAs’ biosynthesis were intermingled with 38 candidate TFs that are predicted to regulate nitrogen uptake. Eventually, 10 candidate TFs were crosslinked, including *HSFB2b* (SMILT000752.1), *LBD12* (SMILT004403.1), *ERF1A* (SMILT009014.1), *ERF98* (SMILT008268.1), *LBD25* (SMILT008917.1), *HSF24* (SMILT012063.1), *RAM1* (SMILT012407.1), *HSFA4B* (SMILT014859.1), *TCP8* (SMILT019331.1), and *WRKY24* (SMILT020775.1).

### 2.10. Validation of Differential Expressed TFs by qRT-PCR Detection

**Figure 8:**
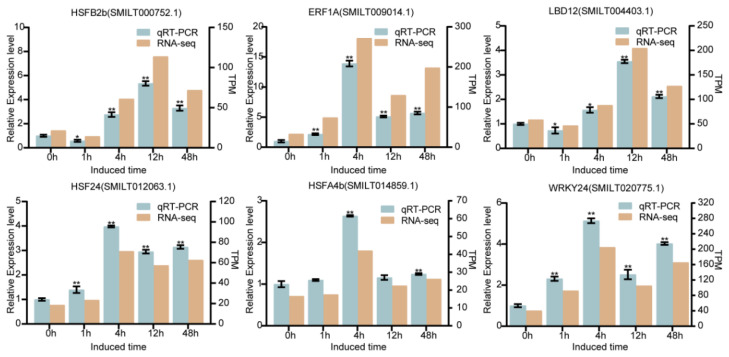
qRT-PCR validation of DEGs in RNA-seq dataset. Expression of 6 TFs related to TAs’ and PHAs’ biosynthesis in response to LN induction using qRT-PCR detection and comparison of their expression with RNA-seq dataset. Error bars denote standard error (±SD). All the detections were triply repeated, * *p* < 0.05; ** *p* < 0.01.

qRT-PCR detection was introduced to verify the differentially expressed TFs obtained by the transcriptome dataset. Six TFs including *HSFB2b* (SMILT000752.1), *LBD12* (SMILT004403.1), *ERF1A* (SMILT009014.1), *HSF24* (SMILT012063.1), *HSFA4B* (SMILT014859.1), and *WRKY24* (SMILT020775.1) possibly modulating TAs’ and PHAs’ biosynthesis under LN induction were chosen for qRT-PCR detection. As shown in Figure 8, the expression pattern of the six TFs detected by qRT-PCR was consistent with those data obtained by transcriptome dataset (Table S13). Through correlation analysis, a nice coherence between the two datasets was observed, with the correlation coefficient ranging from 0.91 to 0.99 (Figure S4). Thus, it is implied that the transcriptome is credible for mining the candidate TFs related to TAs’ and PHAs’ biosynthesis in response to LN induction.

## 3. Discussion

Nitrogen, as an essential nutrient element, exerts its irreplaceable function in modulating plant growth and development, even affecting quality and yield [33,34,35,36,37,38,39,40,41]. How to improve the utilization efficiency of nitrogen has positive significance for green and efficient production of *S. miltiorrhiza*. However, this research item is relatively poor in *S. miltiorrhiza*. To efficiently mine the candidate transcription factors involved in TAs’ and PHAs’ biosynthesis in response to LN stress in *S. miltiorrhiza*, the high throughput sequencing technique was introduced to mine the candidate TFs between the induced groups (1, 4, 12, and 48 h) and the Mock (0 h). In total, 571 TFs were first chosen from the transcriptome dataset of *S. miltiorrhiza* hairy roots, and the number of up-regulated TFs (325) was more than the TFs exhibiting a down-regulation pattern (246) (Table S8).

Co-expression network analysis of gene combinations is thought to be a reliable strategy to mine the candidate TFs in many plant species [42,43,44]. Through co-expression association analysis, 15 *Dof* genes were validated to co-express with 7 PHA biosynthetic genes, and 15 other *Dofs* were dissected to exhibit a co-expression relationship with 6 TA biosynthetic genes in *S. miltiorrhiza* [45]. In *Perilla frutescens*, in order to mine the candidate TFs involved in modulating terpene synthesis, several *PfTPS* genes were employed to construct a co-expression network with up-regulated TFs in the transcriptome dataset [46]. Therefore, in this study, through co-expression analysis, 39 TFs were verified to co-express with TAs’ and PHAs’ biosynthetic genes. Among them, two TFs including *MYB62* (SMILT001326.1) and *NAC2* (SMILT023575.1) were previously reported to regulate TAs’ and PHAs’ biosynthesis by genetic transformation and biological experimental validation in *S. miltiorrhiza* [47,48]. It is noteworthy that nine TFs including *WRI2* (SMILT003037.1), *LBD25* (SMILT008917.1), *ERF20* (SMILT009843.1), *RAM1* (SMILT012407.1), *RHL1* (SMILT015592.1), *CRF3* (SMILT023065.1), *ERF61* (SMILT027065.1), *HSFB3* (SMILT028923.1) and *RSL4* (SMILT032163.1) are newly identified and their biological functions are unclear. Through sequence similarity alignment, five TFs including *MYB62* (SMILT001326.1), *WRKY75* (SMILT004863.1), *MYB20* (SMILT005518.1), *TCP8* (SMILT019331.1) and *MYB82* (SMILT010193.1) were identified to share a high sequence homology with *A. thaliana* and other medicinal herbs, of which their functions were validated to regulate the biosynthesis of secondary metabolites [49,50,51,52,53,54]. In *A. thaliana, AtMYB6*-overexpressing plants exhibited a characteristic of a gibberellic acid (GA)-deficient phenotype, which was partially reversible through exogenous GA application. In *AtMYB62*-overexpressing plants, the expression of several phosphonium ion (Pi) starvation-induced (PSI) genes was suppressed, leading to a decrease in total Pi content in shoots [49,50]. *AtMYB20* was validated to negatively regulate the expression of the type 2C serine/threonine protein phosphatase gene to enhance salt tolerance [51]. Moreover, *AtTCP8* was revealed to directly bind to and activate the promoters of key transcriptional regulators *BZR1* and *BZR2/BES1* involved in brassinolide (BR) signaling, thereby improving Pi uptake in the *A. thaliana* plant [52]. *HlWRKY1*, as a lupulin gland-specific transcription factor and exhibiting high sequence similarity with *AtWRKY75* of *A. thaliana*, could activate the set of promoters driving the genes involved in anthohumol and bitter acids’ biosynthesis in *Humulus lupulus* L., such as chalcone synthase H1, valerophenone synthase, prenyltransferase 1, 1L and 2 and O-methyltransferase-1 [53]. The expression of *MYB82* was suppressed by overexpression of miR828 in *Brassica rapa* plants, and the anthocyanin content as well as the transcript levels of genes related to anthocyanin biosynthesis were decreased, of which the genes include *PAL*, *CHS*, *CHI*, *F3H*, *F3′H*, *DFR*, and *LDOX* [54]. The above studies provide a valuable reference to dissect the underlying biological function of the 39 selected TFs in future research.

In rice, overexpression of ammonium transporter (AMT) genes (*AMT1* and *AMT2*) and glutamine synthetase (GS) genes (*GS1* and *GS2*) can improve ammonium absorption efficiency and boost grain yield [41]. NRT1 and NRT2, as the two dominant transport protein families, are mainly responsible for nitrate uptake [55]. Ammonium nitrogen (NH_4_^+^) and nitrate nitrogen (NO_3_^−^) are the main forms of nitrogen uptake by plants from soil. Nitrate absorbed by plant roots can be reduced to ammonium by nitrate reductase (NR) and nitrite reductase (NiR), by which the ammonium nitrogen is further assimilated through the GS/GOGAT cycle and then participates in the plant proteins’ biosynthesis [56]. The poplar transcription factor *PdGNC* has been demonstrated to regulate nitrogen use efficiency by regulating the expression of several genes involved in nitrogen uptake, including *PdNRT1*, *PdNRT2.4b*, *PdNiR*, and *PdGS* [57]. In the present study, to mine the candidate TFs in response to LN stress, nine marked genes including *Fd-GOGAT*, *NRT2.5*, *NRT1.1*, *GS1.1*, *NiR*, *NRT3.1*, *GS1.3*, *NRT2.7*, and *AMT1.1*, playing vital roles in nitrogen uptake, were employed as the bait to construct the co-expression network with 375 up-regulated TFs. In total, 38 TFs were validated to co-express with the above 9 genes related to nitrogen uptake, with the correlation coefficient >0.9 as the cutoff (Figure 6b, Table S10). Among of the 38 TFs, 21 members were annotated as the WRKY, MYB, bHLH, and AP2-EREBP families, and this number is larger than other TF families (Figure 6). As reported in *A. thaliana*, through the network of a one-hybrid yeast network for nitrogen-associated metabolism (YNM) and experimental validation, MYB73, TCP8, LBD4, and TGA3 were uncovered to be capable of binding to the promoters of genes involved in more than one nitrogen-associated metabolism and growth process [58]. With high sequence similarity with the above four TFs in *A. thaliana*, in present study, the four retrieved TFs including *MYB73* (SMILT006582.1), *TCP8* (SMILT019331.1), *LBD4* (SMILT021236.1), and *TGA3* (SMILT027373.1) might play similar functions in *S. mitiorrhiza*.

By integrating the 39 selected TFs that were predicted to regulate TAs’ and PHAs’ biosynthesis and 38 TFs involved in nitrogen uptake, in total, 10 TFs that might not only regulate TAs’ and PHAs’ biosynthesis but also promote nitrogen uptake were obtained. qRT-PCR detection was further introduced to examine the expression profiles of the above six TFs exhibiting the highest correlation coefficient compared to the four other TFs. It was found that the six TFs were all intensively induced by LN stress, of which we obtained a nice collinear relationship with the RNA-seq dataset. Among the 10 retrieved TFs, there were 3 members annotated as the HSF family, 2 in the AP2/EREBP family, and 2 in the LBD family, and the remaining 3 members were in the GRAS, TCP, and WRKY families, respectively. Among them, *ERF1A* (SMILT009014.1), *HSF24* (SMILT012063.1), and *ERF98* (SMILT008268.1) have been reported to be associated with salt stress in *A. thaliana* and other medicinal herbs [59,60,61]. *OsLBD37/38/39*, belonging to the LBD family, were recognized as key TFs involved in regulating nitrate uptake in *O. sativa* L. [62]. Sharing the highly homologous sequence with the mining *RAM1* (SMILT012407.1) gene, in Medicago, the GRAS family member *RAM1* has been demonstrated to be induced by the phosphate starvation response 2 (*PHR2*) gene related to phosphorus signaling [63]. With high sequence homology to the obtained WRKY24 (SMILT020775.1), AtWRKY33 has been demonstrated to interact with PHR1 to elevate the expression of the *DFR* gene under phosphorus deficiency, thereby enhancing anthocyanin accumulation in *A. thaliana* [64]. Low phosphorus stress has also been validated to induce the expression of *AtWRKY75* in *A. thaliana* [65]. In *Vitis vinifera* L., LN stress can regulate the secondary metabolites’ biosynthesis, including terpenes and phenylpropanoids [66]. As the two most important nutrient elements, nitrogen and phosphorus balance with each other to promote plant growth and development [67,68]. Therefore, it would be an interesting research item to uncover the underlying molecular mechanism of how the 10 retrieved TFs participate in plant nutrient uptake and metabolites’ biosynthesis in *S. miltiorrhiza*.

In summary, the present study has mined not only potential TFs related to TAs’ and PHAs’ biosynthesis but also candidate TFs involved in LN stress. The expression profiles of six candidate TFs induced by LN stress were further detected by qRT-PCR. This work provides effective data for dissecting the underlying molecular mechanism of how candidate TFs regulate TAs’ and PHAs’ biosynthesis under the induction of LN and for breeding a new variety with excellent nitrogen utilization efficiency.

## 4. Materials and Methods

### 4.1. Plant Material and Stress Treatment

Each 1 g of *S. miltiorrhiza* hairy roots was added to 9 conical flasks containing 100 mL of 1/2 Murashige and Skoog (MS) liquid medium, respectively. The flasks were then cultivated in a shaker at 25 °C and 120 rpm for a duration of 40 d. The hairy roots with primary culture were collected, washed with sterilized water, and blotted dry using sterilized paper. Three bottles of hairy roots were set as a biological replicate group (Figure S5). All 9 bottles of hairy roots were transferred to 1/2MS liquid basal medium containing one-twenty of the NO_3_^−^ compared to the normal usage. Half of the hairy roots at 0, 1, 4, 12, and 48 h time intervals were individually collected, while the other half of the samples were gathered after another induction period of 7 d [15]. All the collected hairy roots were quickly frozen in liquid nitrogen and subsequently stored at −80 °C for further detection of the gene expression and tanshinone and phenolic acid contents with three technical replicates.

### 4.2. Measuring the TA and PHA Content

Hairy roots were dried in an oven and ground into a fine powder with a mortar and pestle. Each 50 mg of powder sample was subjected to extraction of TAs using 15 mL of methanol/dichloromethane (3:1, *v*/*v*), sonicated for 1 h (KunshanKQ-500B, Kunshan UL transonic instruments, Kunshan, China), and centrifuged at 25 °C to obtain the supernatant extract. The primary extract was then evaporated with a rotary evaporator to obtain dry extract powder, dissolved in 2 mL of methanol and filtered by a 0.22 µm 3M membrane. Then, high-performance liquid chromatography (HPLC) was introduced to measure the TA content including dihydrotanshinone (DT), cryptotanshinone (CT), tanshinone I (TI), tanshinone IIA (TIIA), and total of tanshinones (TT). The column oven temperature was 20 °C and the detection wavelength was set at 270 nm [26].

To quantify the PHA content consisting of caffeic acid (CA), rosmarinic acid (RA), salvianolic acid (SalB) and total of phenolic acid (TPA) by HPLC, about 50 mg of plant powder was weighed, put into a 2 mL centrifuge tube, and then subjected to ultrasound with 2 mL of 80% methanol for 1 h (KunshanKQ-500B, Kunshan UL transonic instruments, Kunshan, China). The solution was centrifuged with 5000× rpm for 2 min to obtain the supernatant (Eppendorf, Hauppauge, NY, USA) and then filtered with a 0.22 µm filter membrane to obtain the PHA extract to conduct HPLC detection with a column oven temperature of 35℃ and a detection wavelength of 281 nm [26].

### 4.3. RNA Isolation, cDNA Library Preparation, and Sequencing

Total RNA from 15 hairy root samples was independently extracted using QIAzol Lysis Reagent (Qiagen, Dusseldorf, Germany) as described previously. The concentration and purity of the obtained RNA were examined by Nanodrop2000 (Thermo Fisher Scientific, Waltham, MA, USA). The integrity of the extracted RNA was monitored using agarose gel electrophoresis analysis. By using magnetic beads with Oligo (dT) to match with the ployA tail, the mRNA was further purified from total RNA. In total, 1 µg of total purified mRNA was introduced to conduct transcriptome library construction and sequencing with the Illumina NovaSeq X Plus platform. All the transcriptome sequencing datasets were deposited in the Genome Sequence Archive held by the Beijing Institute of Genomics Data Center (https://bigd.big.ac.cn/), Chinese Academy of Sciences, for preparing the cDNA library, as described previously [69].

### 4.4. Data Filtering and De Novo Assembly

After transcriptome sequencing, the adapter sequences, repeated, and low-quality reads were filtered to obtain clean reads and a high-quality RNA-sequencing dataset of *S. miltiorrhiza*. On the basis of the existing reference genome [70], mapped reads were assembled and spliced using the Cufflinks software (https://cole-trapnell-lab.github.io/cufflinks/, accessed on 3 July 2024) [71] and StringTie (http://ccb.jhu.edu/software/stringtie/, accessed on 3 July 2024) [72].

### 4.5. Functional Annotation and Classification

Diamond and HMMER were employed to search for assembled transcripts in six public databases of NR, GO, KEGG, Pfam, EggNOG, and Swiss-Prot with an E-value of 1 × 10^−5^ as the cutoff. Annotations of DEGs with the highest hit rate were gathered to form an annotation list (Supplementary Table S3).

### 4.6. Enrichment of Differentially Expressed Genes

To explore differential expression genes (DEGs) between the Mock (0 h) and each induced group (1, 4, 12 and 48 h), the expression level of each transcript was calculated by the Transcripts Per Million mapped reads (TPM) method. The RSEM software, version 1.3.3, was introduced to quantify gene abundances [73]. Essentially, DEGs were mined using DESeq2, version 1.24.0, with |log_2_FC| ≥ 1 and *p*-adjust < 0.05 (DESeq2) as the cutoff [74]. Moreover, GO and KEGG enrichment analyses were performed to identify DEGs at the Bonferroni-corrected *p*-value < 0.05 compared with the whole-transcriptome background by the Goatools (version 0.6.5) and Python (version 3.10) scipy software, respectively.

### 4.7. Co-Expression Analysis

For the DEG assay, TPM values were used to normalize the gene expression levels. Statistical comparison of TPM values between the Mock and each treatment group was conducted using the Bowtie2 software (version 2.4.1). Statistical analysis was examined using the FDR method with the corrected *p*-value of 0.05 as the selected threshold. The co-expression network between the candidate biosynthetic enzyme genes involved in TAs’ and PHAs’ biosynthesis and nitrogen uptake genes was constructed with the weighted gene co-expression network analysis (WGCNA) method. Co-expression correlation was defined with *p*-adjust < 0.05. The heat map was generated by the TBtools software (version 2.056).

### 4.8. Quantitative Real-Time PCR Detection

Primer pairs for qRT-PCR detection were designed by Primer 5.0 using the unigene sequences obtained in this study. First-strand cDNA was generated from 100 ng of total RNA using a reverse transcriptase (Vazymes, Nanjing, China). The cDNA was diluted 10-fold. Real-time PCR was conducted using the 2×Taq Pro Universal SYBR qPCR Master Mix (Vazymes). The gene expression level was normalized to the control gene of *SmActin* by the 2^−ΔΔCT^ method. The data presented represent the mean of three independent biological replicate samples.

## 5. Conclusions

In the present study, a transcriptome sequencing database derived from 15 *S. miltiorrhiza* hairy root samples together with gene co-expression analysis was employed to mine candidate TFs modulating TAs’ and PHAs’ biosynthesis under LN stress. In total, 10 TFs including *HSFB2b*, *LBD12*, *ERF1A*, *ERF98*, *LBD25*, *HSF24*, *RAM1*, *HSFA4B*, *TCP8*, and *WRKY24* were retrieved and qRT-PCR analysis was introduced to further validate the expression profile of candidate TFs under LN stress. This study lays a research foundation to dissect, in-depth, the molecular regulatory network of candidate TFs to improve nitrogen utilization efficiency and medicinal substances’ biosynthesis, of which it is helpful to breed new *S. miltiorrhiza* germplasm resources through genetic engineering manipulation.

## Figures and Tables

**Table 1:** Summary of transcripts induced by different times after LN stress in *S. miltiorrhiza*.

Sample	Raw Reads	Raw Bases	Clean Reads	Clean Bases	Q30 (%)	GC Content (%)
0h_1	41,996,116	6,341,413,516	41,747,548	6,277,251,062	96.86	48.83
0h_2	44,891,932	6,778,681,732	44,612,244	6,711,502,350	96.78	49.01
0h_3	44,646,604	6,741,637,204	44,364,466	6,675,923,766	96.77	49.12
1h_1	41,331,902	6,241,117,202	41,086,500	6,187,491,240	96.65	48.67
1h_2	47,329,878	7,146,811,578	47,054,614	7,083,282,107	96.91	48.6
1h_3	42,129,134	6,361,499,234	41,895,170	6,287,847,014	96.72	48.74
4h_1	41,322,204	6,239,652,804	41,072,752	6,160,922,097	96.69	48.10
4h_2	43,243,950	6,529,836,450	42,999,830	6,462,453,273	96.91	48.50
4h_3	46,512,196	7,023,341,596	46,232,832	6,946,228,890	96.89	48.87
12h_1	43,748,030	6,605,952,530	43,488,554	6,535,604,044	96.93	48.80
12h_2	44,072,088	6,654,885,288	43,828,444	6,595,059,343	96.93	48.42
12h_3	44,827,100	6,768,892,100	44,562,632	6,700,988,682	96.93	48.73
48h_1	40,305,996	6,086,205,396	40,060,196	6,026,652,901	96.88	48.54
48h_2	44,159,486	6,668,082,386	43,814,930	6,587,029,688	96.12	48.95
48h_3	43,280,274	6,535,321,374	43,000,430	6,468,945,750	96.77	48.89
Total	653,796,890	98,723,330,390	649,821,142	97,707,182,207	-	-
Average	43,586,459	6,581,555,359	43,321,409	6,513,812,147	96.78	48.72

## Data Availability

All data generated or analyzed during this study are included in this published article and its Supplementary Materials.

## Supplementary Materials

The following supporting information can be downloaded at: https://www.mdpi.com/article/10.3390/ijms26041774/s1.

## Abbreviations

The following abbreviations are used in this manuscript:

TAs	Tanshinones
PHAs	Phenolic acids
MVA	Mevalonate
MEP	2-C-methyl-D-erythritol 4-phosphate
IPP	Isopentenyl diphosphate
DMAPP	Dimethylallyl diphosphate
IDI	Iso-pentenyl diphosphate isomerase
GGPPS	Geranylgeranyl pyrophosphate synthase
GGPP	Geranylgeranyl pyrophosphate
RAS	Rosmarinic acid synthetase
RA	Rosmarinic acid
CYP98A14	Cytochrome P450-dependent monooxygenase
TF	Transcription factor
PHH	Post-harvest hardening
DEG	Differential expression gene
qRT-PCR	Quantitative real-time PCR
DT	Dihydrotanshinone
CT	Cryptotanshinone
TIIA	Tanshinone IIA
TT	Total tanshinones
Sm	*Salvia miltiorrhiza*
TPA	Total of phenolic acids
CA	Caffeic acid
SalB	Salvianolic acid B
PCA	Principal component analysis
BP	Biological process
CC	Cellular component
MF	Molecular function
TPM	Transcripts Per Million
AMT	Ammonium transporter
GS	Glutamine synthetase
NR	Nitrate reductase
NiR	Nitrite reductase
GCN	General control non-derepressible
MS	Murashige and Skoog
HPLC	High-performance liquid chromatography
WGCNA	Weighted gene co-expression network analysis
