# Supplementary material for: Mining the Candidate Transcription Factors Modulating Tanshinones’ and Phenolic Acids’ Biosynthesis Under Low Nitrogen Stress in Salvia miltiorrhiza
Source: Int J Mol Sci. 2025 Feb 19;26(4):1774. doi: 10.3390/ijms26041774 (PMC11855394; doi:10.3390/ijms26041774)
Supplement: Supplementary file 1 [file ijms-26-01774-s001.zip › ijms-3448843-supplementary.pdf]

Supplementary Figures

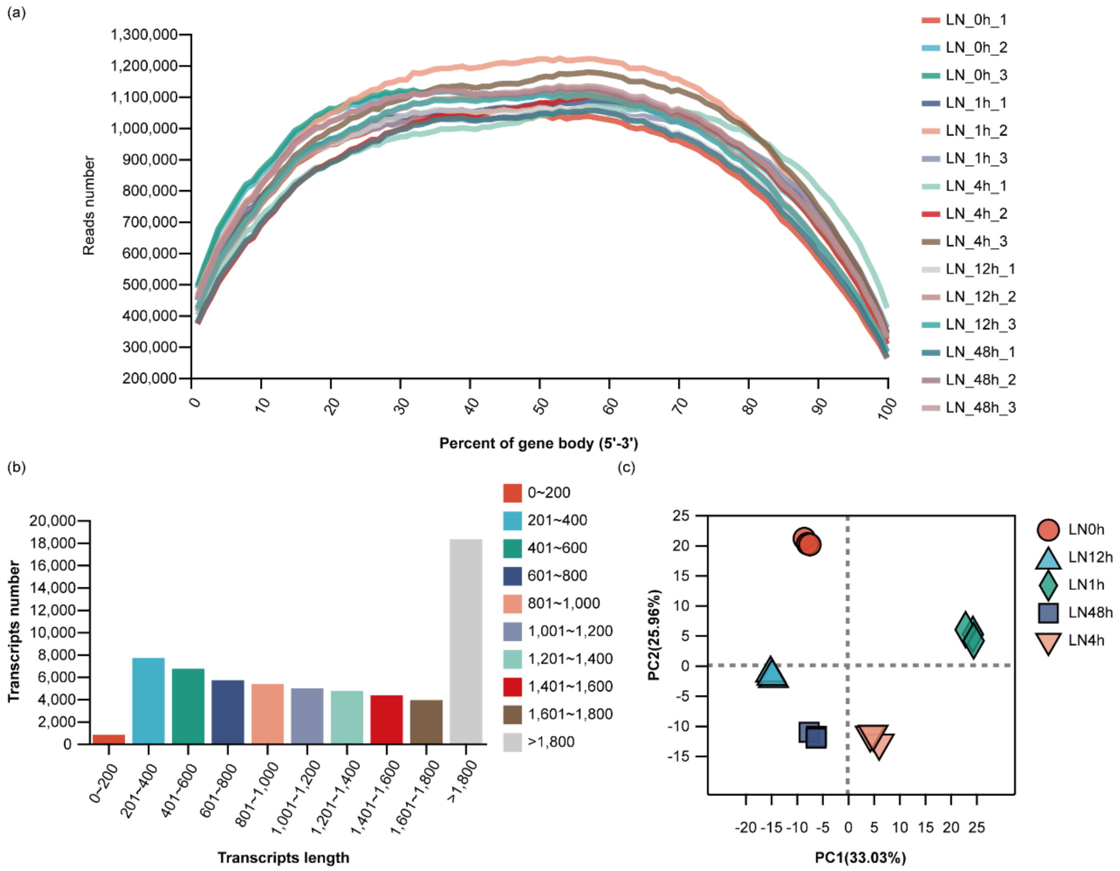

Figure S1. Transcriptome assembly.(a) Coverage of sequencing. The horizontal coordinate is the base length of a single gene as a percentage of the total base length, with 0 indicating the 5′ end of a gene and 100 indicating the 3′ end of a gene; the vertical coordinate is the sum of the number of sequence entries in the corresponding intervals from the horizontal axis positions of all the genes compared to the sum of the sequence entries in the corresponding intervals. (b) Length distribution of transcripts.The horizontal coordinate is the range of transcript lengths; the vertical coordinate is the number of transcripts within that range of transcript lengths. (c) PCA analysis. The distance of each sample point represents the distance of the samples, with closer distances indicating higher similarity between the samples.

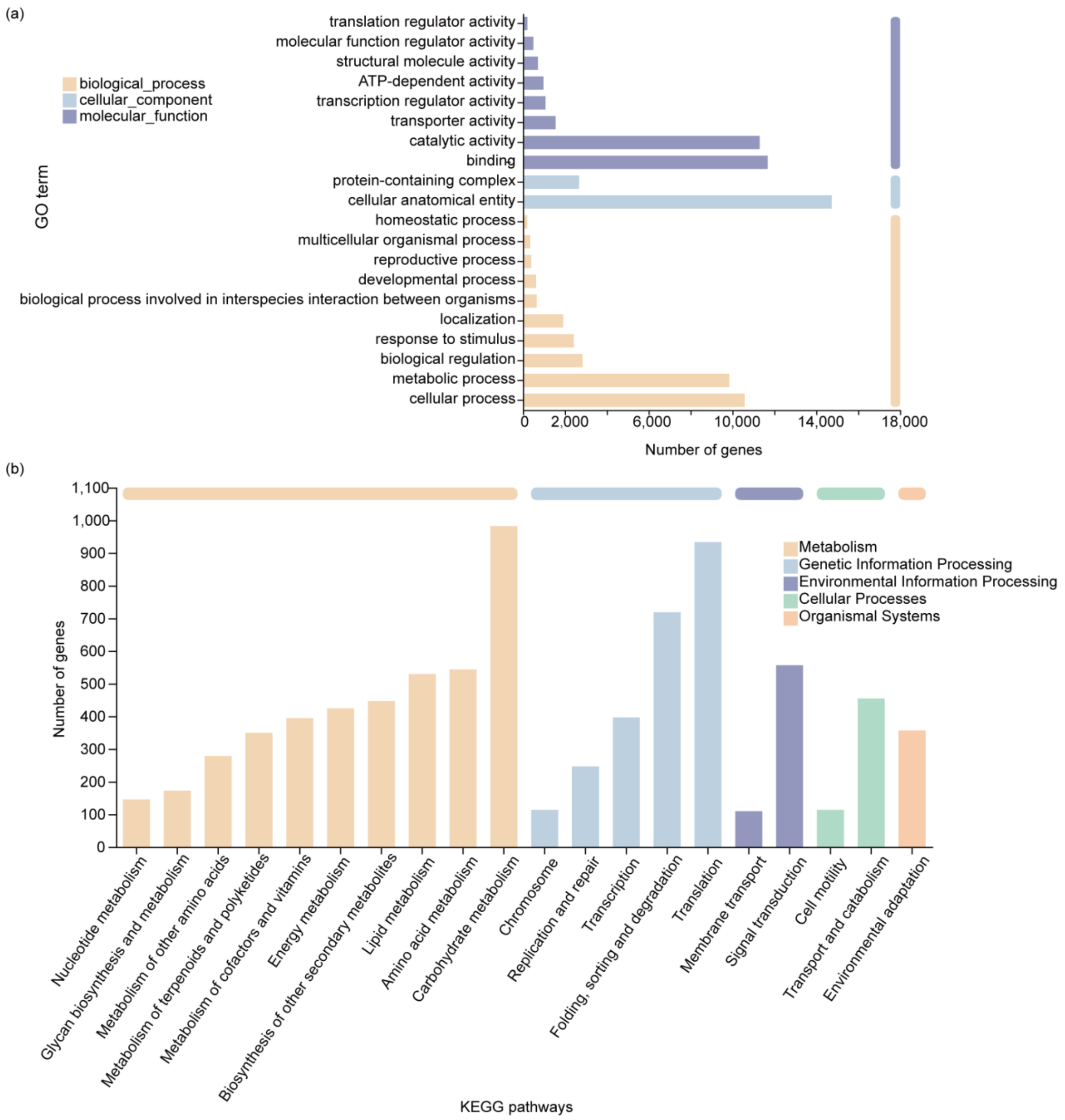

Figure S2. Functional annotation of unigenes.(a) GO annotation. The results were classified into three main categories: biological process, cellular component, and molecular function. The x-axis represents the number of unigenes and the y-axis represents the GO functional category. (b)KEGG annotation. The y-axis represents the number of unigenes and the x-axis represents the KEGG functional categories.

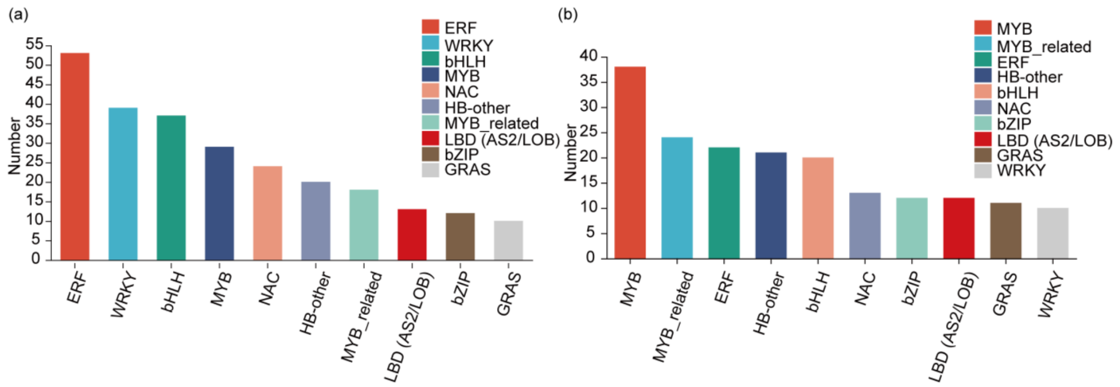

Figure S3. Differentially expressed transcription factors.(a) Up-regulated transcription factors. (b) Down-regulated transcription factors.

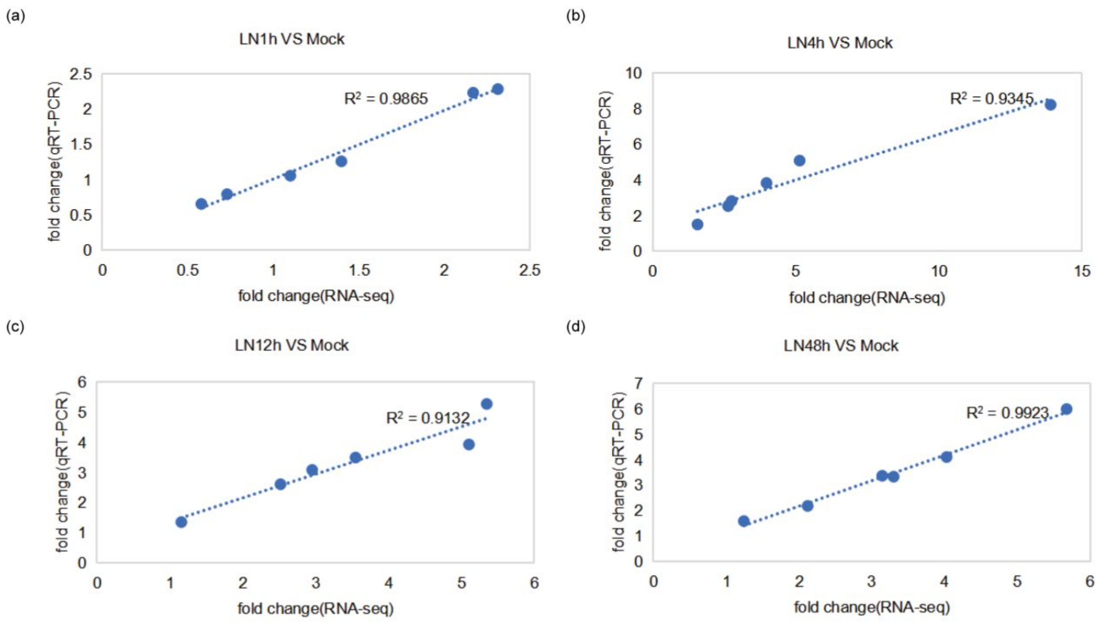

Figure S4. Correlation of expression profiles of 6 TFs obtained from qRT-PCR detection and RNA-seq.

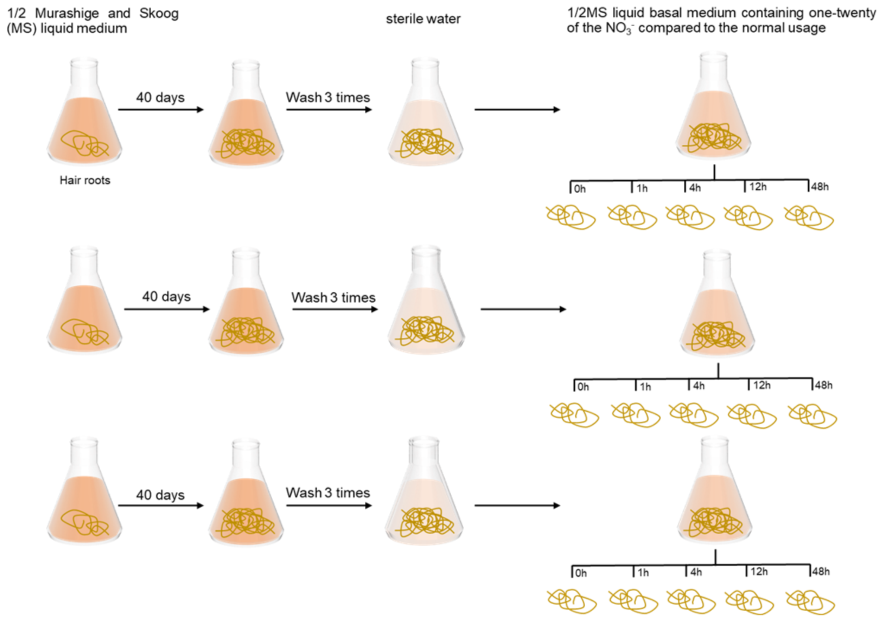

Figure S5. Flowchart for low nitrogen treatment of *S. miltiorrhiza* hairy roots.
